# Isolated Otologic Involvement of IgG4 Related Disease: A Case Report and Review of Literature

**DOI:** 10.7759/cureus.23787

**Published:** 2022-04-03

**Authors:** Hugh P Mallany, Vincent Anagnos, Tiffany Hwa, Tiffany Chao, Kathleen Montone, Steven Eliades

**Affiliations:** 1 Otorhinolaryngology, University of New England, Biddeford, USA; 2 Otorhinolaryngology, Perelman School of Medicine at the University of Pennsylvania, Philadelphia, USA; 3 Pathology and Laboratory Medicine, Perelman School of Medicine at the University of Pennsylvania, Philadelphia, USA

**Keywords:** sigmoid sinus thrombosis, autoimmune disease, mastoiditis, hearing loss, igg4 related disease

## Abstract

Immunoglobulin G4 related disease (IgG4-RD) is a systemic autoimmune disease process that classically presents with multi-organ involvement; however isolated involvement of various structures within the body has also been described. Histopathologic examination is considered the gold standard for diagnosis. Glucocorticoids are well established as first-line treatment, but relapses are common, and consultation with rheumatology, immunology, and/or oncology teams is almost always warranted for proper medical management and disease maintenance. Given the relative infancy of IgG4-RD as an accepted diagnosis and the overall rarity of the disease, much still needs to be learned about this variable disease process. We present this case of isolated otologic and lateral skull base involvement of IgG4-RD to contribute to the understanding of this exceedingly rare clinical entity.

## Introduction

Immunoglobulin G4 related disease (IgG4-RD) is a systemic autoimmune disease that typically involves multiple organ systems, including the pancreas, lungs, and liver [1]. While involvement within the head and neck region is relatively rare, conditions such as Mikulicz disease, Riedel's thyroiditis, and chronic sclerosing sialadenitis are all well-characterized IgG4 related processes [2]. Additionally, isolated otologic involvement of IgG4-RD is incredibly rare as only a few case reports are available in the literature [3,4]. In this article, we report a case of a 26-year-old female with a history of recurrent left-sided middle ear infections who presented to a tertiary care hospital with acutely worsening hearing loss, otalgia, and headache. Computer tomography (CT) of the temporal bone showed evidence for coalescent mastoiditis with sigmoid sinus thrombosis. She was taken urgently to the operating room for mastoidectomy. Upon entering the mastoid cavity, a tan rubbery friable material appeared to be filling the mastoid cavity without signs of infection or purulence. Microbiological and histological analysis demonstrated plasma cell infiltrates with elevated plasma IgG4 further confirming suspicions of IgG4-RD of the lateral skull base. Further workup and whole-body imaging failed to definitively find any other involved organ systems. This case report and review of the literature is intended to provide further insight into IgG4-RD and its potential to present as an isolated disease process within the head and neck region and specifically within the lateral skull base.

## Case presentation

**Figure 1:**
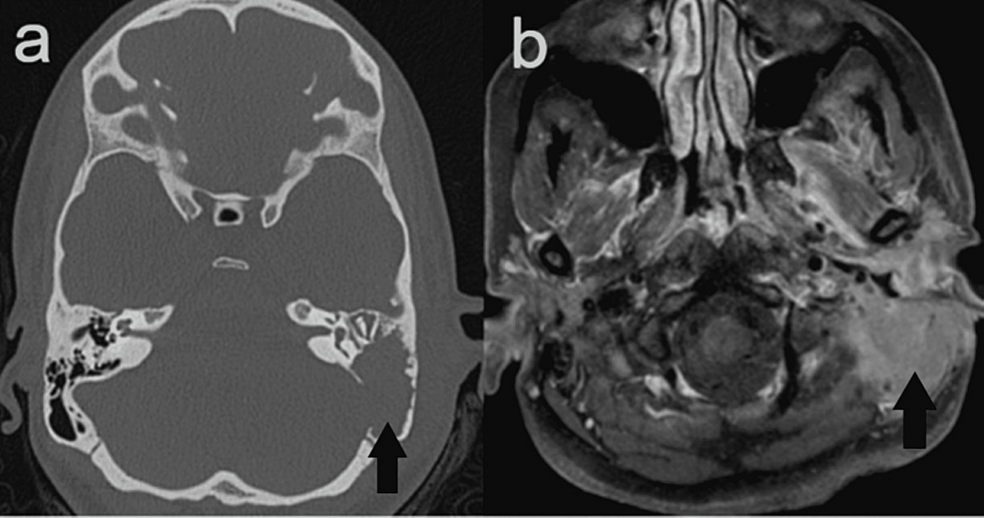
CT of the temporal bone (A) and MRI of the brain (B) A. Axial view of CT scan showing coalescent process involving the left temporal bone. B. Axial view of T1-weighted MRI demonstrating a solidly enhancing, mass-like lesion centered within the mastoid segment of left temporal bone, with associated osseous destruction, extending into the left periauricular soft tissues, left external auditory canal, and minimally into the epidural space along the inferior aspect of the left temporal lobe.

A 26-year-old female with a history of progressive left ear pain and subjective hearing loss over the past two months presented to our tertiary care center. In the proceeding two months, the patient was managed for otitis media with pressure equalizing tubes, ciprodex otic drops, and trials of oral augmentin and ciprofloxacin. Physical exam upon arrival demonstrated significant pain with otoscopy, facial movement full and equal bilaterally, and moist yellow debris found filling the external auditory canal making tympanic membrane visualization difficult on the left side. There was no mastoid tenderness or overlying erythema, or edema. The remainder of the physical exam, vital signs, and laboratory values were unremarkable. A computer tomography (CT) of the temporal bone and magnetic resonance imaging (MRI) of the brain demonstrated a coalescent left mastoiditis with pachymeningeal enhancement seen adjacent to the tegmen mastoideum. Abnormal, asymmetric low T2 signal within the left sigmoid sinus also demonstrated evidence for dural venous sinus thrombosis. There were no signs of intracranial infection (Figure 1).

**Figure 2:**
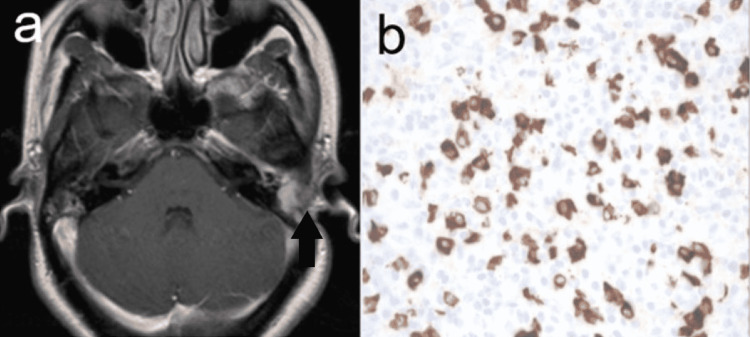
MRI of the brain (A) and histopathologic analysis of excised mass (B) A. T1-weighted MRI showing resolution of temporal mass. B. Histopathologic analysis of excised mass with IgG4 immuno-stain. IgG4 cells are stained brown

The patient was then taken to the operating room urgently for mastoidectomy with the presumed diagnosis of acute coalescent mastoiditis. Intraoperatively, there was near complete erosion of the mastoid cortex with an associated tan, friable, rubbery tissue lesion that encompassed the majority of the mastoid cavity without any signs of acute infection or purulence. Complete debridement of the mastoid cavity was attempted, but the soft tissue lesion was noted to be tightly adherent to the sigmoid sinus and tegmen, so subtotal resection was deemed appropriate pending a final diagnosis. Histopathological analysis demonstrated mixed inflammatory cell infiltrate and a large number of polyclonal CD138+ cells with IgG4 plasma cells comprising approximately 40% of total plasma cells within the tissue sample. Quantitative IgG subclass analysis also showed a mild increase in IgG4 isotype at 125mg/dL (normal 1-123mg/dL) (Figure 2). The combination of these findings was consistent with a presumed diagnosis of IgG4-RD. The patient was then started on high dose prednisone of 1mg/kg, daily and therapeutic anticoagulation for sigmoid sinus thrombosis per rheumatology recommendations.

Repeat post-operative imaging at nine months follow-up demonstrated a significant reduction in the size of previous lesions in the mastoid bowl, middle ear, and horizontal petrous ridge. Intradural pachymeningeal thickening and enhancement in the posterior cranial fossa seen in previous studies were also improved. More importantly, the patient’s symptoms of ear pain and hearing have improved dramatically.

## Discussion

In this case, we report a 26-year-old woman who presents with subjective hearing loss, otalgia, and recurrent otitis media who underwent mastoidectomy for coalescent mastoiditis seen on CT. Mastoiditis, in this case, was presumed to be caused by IgG4-RD. While there are no official diagnostic criteria for IgG4-RD, several common criteria have been used within the literature as an outline for what may constitute a diagnosis of IgG4-RD. Most commonly a tissue diagnosis of >30 IgG4 plasma cells per high powered field is the most widely accepted criteria for inclusion. Also, elevated serum IgG4 levels can assist in the workup; however, up to 30% of these patients have normal levels of serum IgG4 [1,5,6]. Treatment for IgG4-RD typically involves surgical biopsy or debridement followed by systemic immunosuppressive therapy. The first-line therapy with high-dose glucocorticoids seems to be well established [5,6,7-9]. Also, other immunosuppressive and chemotherapeutic treatments have been utilized, including cyclophosphamide and rituximab. Additionally, it is important to consider a milder version of IgG4-RD known as myofibroblastic inflammatory tumor (IMT). While these conditions are similar entities, there are significant differences between the two. In an analysis of reported cases, 64% of individuals with IMT had ALK gene mutations present in tissue samples. Furthermore, of 36 inflammatory myofibroblastic tumors, 15 cases showed an IgG4/IgG ratio ≥0.10, which falls just shy of the commonly used cut-off for IgG4 sclerosing disease as seen in our case, making this an extremely likely differential [10]. Several cases also demonstrate coalescent mastoiditis with accompanying sigmoid sinus thrombosis, a feature shared with this case as well. Furthermore, the patient experienced continued hearing loss at a five-week follow-up which is not unexpected. Improvement or remission of the disease process generally occurs once the systemic treatment is initiated; however, several months of persistent therapy is often required in order to see objective improvement on the audiogram [3,7]. Outcomes of immunosuppressive treatment are generally efficacious, with a narrowing of an air-bone gap of 40dB seen within several months following initiation of treatment [7].

## Conclusions

IgG4 related disease (IgG4-RD) is a rare clinical entity, and our understanding of this disease process is continuously evolving as more information is presented from various case presentations. However, its discovery is now giving explanations and potential treatment options for diseases not previously well understood. As more cases are reported, clinical guidelines and algorithms should be considered to properly manage these patients. Additionally, with the increasing use of biological agents, this disease process should be investigated for its efficacy with those types of therapy due to the immunologic basis of the condition. It is in the interest of the otolaryngologist to keep this rare process on the differential diagnosis in cases of atypical and refractory otologic complaints, even with the lack of other systemic findings.
